# Imagine: Design for Creative Thinking, Learning, and Assessment in Schools

**DOI:** 10.3390/jintelligence8020016

**Published:** 2020-04-15

**Authors:** Yigal Rosen, Kristin Stoeffler, Vanessa Simmering

**Affiliations:** ACTNext by ACT, 500 ACT Drive, Iowa City, IA 52243, USA; kristin.stoeffler@act.org (K.S.); vanessa.simmering@act.org (V.S.)

**Keywords:** creativity, creative thinking, assessment, learning technology

## Abstract

Although not generally included in classroom activities of the past, cultivating creative thinking is considered one of the core strands in future-focused learning in schools. Learning focused on creative thinking is uncommon in school, mainly due to a lack of consensus on the definition of the creative thinking competency and a lack of effective methods designed for curriculum-embedded implementations of creative thinking learning and assessment in classrooms. This paper describes the development of a framework for formative assessments of creative thinking frameworks and provides considerations for the design of technology-enhanced learning and assessment in support of creative thinking competency in students. Task models described in the paper aimed to cultivate creative thinking and elicit evidence on competency development in students. Future directions for the development and validation of learning and assessment approaches are discussed.

## 1. Introduction

Cross-cutting higher-order skills, such as creative thinking, transforms lives and drive economies. Creative thinking stands out among the thinking skills due to its increasing importance in the labor market and personal and civic life, and the challenges associated with cultivating creative thinking in schools (Beghetto 2010; Bughin et al. 2018; Grigorenko 2019; Lucas and Spencer 2017; World Economic Forum 2019). Its capacity to facilitate the wave of innovation created by the merging of minds, industries, and fields highlights its potential and value for society. The importance of creative thinking skills specifically is highlighted by the selection of creative thinking as the innovative domain for the 2021 Programme for International Student Assessment (PISA) by the Organisation for Economic Co-operation and Development (OECD 2019). Creative thinking was also highlighted as a critical component of the transformative competencies by the OECD’s Education 2030 initiative (OECD 2018); the trends established by the OECD Centre for Educational Research and Innovation (Vincent-Lancrin et al. 2019); and the essential role of creative thinking in achieving many of the seventeen Sustainable Development Goals outlined by the United Nations (United Nations High Commissioner Transforming Our World: The 2030 Agenda for Sustainable Development 2015). 

The development of an assessment to measure creative thinking requires the ability to parse and define and measure the essential competencies and processes required for creative thinking. Attempts to achieve this type of definition and measurement at scale have been limited, and the most notable work in this space has been achieved through the development of a Creative Thinking Framework and Assessment by the OECD for PISA 2021 (OECD 2019). This paper presents the recent development of a framework for formative assessment of creative thinking. Here we describe development of the research-based competency model; considerations for the design of technology-enhanced learning and assessment tasks; and specific task models that can support the necessary cognitive and affective processing and skill development in schools. Our goal is not to provide a complete review of the literature leading up to this stage, but rather to focus primarily on progress related to PISA and our work building from that framework.

## 2. Competency Model Development

Although most researchers agree that creativity involves the development of a novel product, idea, or problem solution that is of value to the individual and/or the larger social group, researchers have had great difficulty finding a consensus on the definition beyond these two criteria of novelty and value (Kaufman and Baer 2012; Plucker et al. 2004; Runco et al. 2001; Sawyer 2011). Different models place emphasis on various components of the creative process, often focusing on the particular contexts in which they were developed. For example, synectics is an approach to creative problem solving that originally focused on group dynamics in business settings, but can be applied to individuals and other settings (including classrooms) as well (Weaver and Prince 1990). The focal process in synectics is termed “excursion,” which involves: (1) temporarily putting the problem out of mind, also known as incubation; (2) deliberately focusing on seemingly irrelevant information, which should be generated freely by participants; then, (3) force-fitting the irrelevant information and the problem together to invent new ways to connect them. The goal of this force-fit is to bring to light dimensions or approaches to the problem that are not readily apparent at first. The process is most successful when a facilitator moderates interactions among members to discourage statements that discount ideas and encourage generative thinking. 

Amabile’s componential model (Amabile 1983, 2012; Amabile and Pratt 2016) was similarly proposed in a business context to describe both creativity in individuals and innovation in organizations within the same model. The original three components were domain-relevant skills (i.e., knowledge, technical skills, talent), creativity-relevant skills (i.e., cognitive style, heuristics to generate novel ideas, work style), and task motivation (i.e., attitudes, self-perception), as possessed by individuals (Amabile 1983); this model was updated to include organizational components of motivation, resources, and management skills (Amabile and Pratt 2016). In addition to these components, Amabile’s model includes a five-stage creative process—agenda setting; stage setting; producing ideas; testing and implementing ideas; and assessing the outcome—which is influenced by both individuals and the organization. Although this model was developed in the context of workplace innovation, the components and process can be construed analogously in classrooms.

Alternatively, Mumford and colleagues (Mumford et al. 1991; Mumford and McIntosh 2017) proposed a consensual process model that was built on three assumptions. First, creative problem solving is more narrowly construed, requiring high quality, original and elegant solutions to problems that are novel, complex, and ill-defined. Second, problem solving requires specific knowledge or expertise. Third, similar processes underlie creative thought across domains, although there will be domain-specific knowledge requirements with potentially differential weightings of processes across domains. From these assumptions they proposed an iterative eight-stage process model in which earlier steps may be revisited and repeated as needed: problem defining, information gathering, concept selection, conceptual combination, idea generation, idea evaluation, implementation planning, and adaptive execution. The initial focus on the problem in this model highlights the assumption that problems that are not novel, complex, or ill-defined will not need creative solutions. 

As these examples illustrate, there is overlap in key ideas between prominent models of creativity, but there are differences in focus and emphasis for different features and applications. Other distinctions in the literature on creativity are the types of behaviors and applications of interest. Creativity may range from a low level, as in, for instance, solving a typical-insight problem, to a very high level involved in the shift of paradigms or genres involved in science and art. The two-level definition of creativity is widely accepted by researchers, with the two levels referred to as “Big C” and “little-c” (Merrotsy 2013, but see Runco 2014a for a critique). Big-C creativity occurs when a person solves a problem or creates a product that has a major impact on how other people think, feel, and live their lives. This level of creativity consists of clear-cut, eminent creative contributions. For example, the “Big C” creativity that is associated with technology breakthroughs or art masterpieces demands that creative thinking be paired with significant talent, deep expertise, and high levels of engagement in a specific domain of knowledge, along with the recognition from society that the product has value. Little-c creativity, on the other hand, includes actions which a non-expert may use to adapt to changes each day. Little-c or everyday creativity can be achieved by nearly all people capable of engaging in creative thinking, and can be developed through practice and honed through education. Teachers who foster little-c creativity in their classrooms recognize that creative thinking is not an extracurricular activity, but rather an interconnected part of their everyday curriculum, especially when naturally integrated into domain-specific learning and assessment assignments (Beghetto et al. 2014; Grigorenko et al. 2008).

We contextualize our work within the larger arena of creativity research by defining creative thinking as a set of skills used in approaching tasks, problems, and situations in an unconventional way (Kaufman and Baer 2012; Amabile and Pratt 2016; Grigorenko et al. 2008; Rosen and Tager 2013; Sternberg and Lubart 1991). To this definition we add the specification that creative thinking can be taught and developed to the point of competency (e.g., along with specific levels of proficiency) in conjunction with one or more academic domains. Assessing levels of proficiency requires a large-scale validation of the framework and assessment approach to creative thinking, which has not been attempted prior to the current PISA study. According to this approach, a student’s performance in creative thinking tasks is linked to the level of difficulty of the task with specific levels of associated proficiency. Students functioning at that corresponding level of competency can address the associated tasks successfully. Proficiency is quantitatively distributed, along with the difficulty level of the tasks, so that the closer the match between the two distributions, the higher the probability of a successful outcome in approaching a creative thinking task. Data from creative thinking tasks in our validation studies were analyzed using the Rasch and interaction models (Haberman 2007; Maris et al. 2015). The interaction model is an extension of the Rasch model that allows for evaluating the extent to which the Rasch model fits the data, both substantively and statistically.

In line with this approach, creative thinking is defined in the PISA 2021 Creative Thinking Framework as the competence to engage productively in the generation, evaluation, and improvement of ideas that can result in original and effective solutions, advances in knowledge, and impactful expressions of imagination (OECD 2019). The domain is focused on three primary facets: (1) Generating diverse ideas: this focuses on students’ capacities to think flexibly across domains. (2) Generating creative ideas: focuses on students’ capacities to search for appropriate and original ideas across different domains. (3) Evaluating and improving ideas: focuses on students’ capacities to evaluate limitations in given ideas and find original ways to improve them. PISA 2021 will primarily measure the extent to which students can generate diverse and creative ideas when working on open, real-life tasks, and the extent to which they can evaluate and improve upon those ideas. Creative thinking tasks are being designed in relation to “little c” creativity in order to minimize the importance of domain-specific knowledge and artistic or expressive talent for performance and to put a stronger focus on the malleable capacity of individuals to engage in creative thinking as a set of transferrable skills and processes that can be used across domains and to employ a wide range of expertise. Students will explore their ideas and solutions in four different domains: written expression, visual expression, scientific problem solving, and social problem solving. They will thus engage in different activities, such as writing short stories, creating a logo, finding multiple methods to solve an engineering problem, or exploring innovative solutions to complex social issues. 

The limitations of this definition and assessment are rooted in the contextualization of this framework for the educational background and large-scale assessment constraints relevant for 15-year-old students around the world. The conceptualization and assessment make efforts, however, to maintain the critical and transferrable foundations and applications of creative thinking. Most notably, effectively engaging in creative thinking requires an individual to generate, reflect, and iterate on ideas while maintaining a balance of usefulness and novelty. These foundational concepts have been part of models of creativity for decades (Corazza and Agnoli 2016; Feldhusen and Goh 1995) but have not yet led to a unified framework on which large-scale assessments can be built. Prior assessments have been developed and applied at smaller scales, often using self-reported measures for ease of administration and desirable psychometric properties (Snyder et al. 2019). PISA was designed to provide a psychometrically sound large-scale assessment, but to focus on group (country) level reports rather than individual proficiency. Furthermore, many models of creativity (including the framework in PISA) have not addressed learning or development of these skills (Barbot 2019; Bolden et al. 2019), thereby limiting their applicability to foster creative thinking in students.

**Figure 1:**
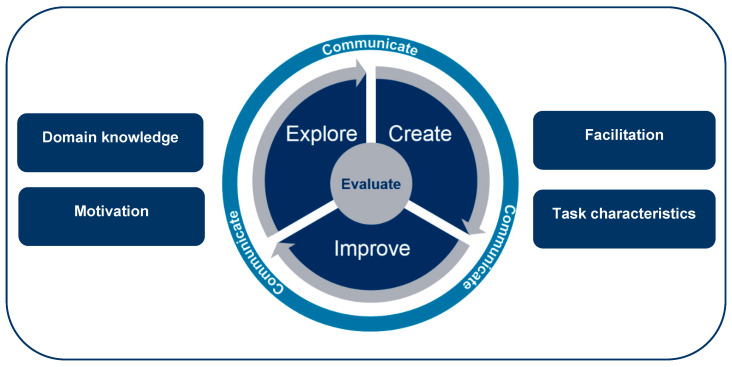
Creative thinking model for learning and assessment.

The proposed expanded Creative Thinking Learning and Assessment Model (see Figure 1) is designed to capitalize on the foundational understanding developed through PISA 2021 work and to build upon insights gained through their application in learning and formative assessment. The goal of formative assessment is to provide students with feedback and guidance based on their current levels of some target competency, and prior research on creative thinking shows that formative assessment is a promising approach to its development (Bolden et al. 2019). Our approach to learning builds on four of the five key formative assessment strategies proposed by Black and Wiliam (2009): clarifying learning intentions and success criteria; engineering effective tasks that elicit evidence of learning; providing feedback that moves learners forward; and activating students as owners of their learning.

In application, this allows for more emphasis to be placed on the evaluative, metacognitive, and communicative processes involved with the creative thinking processes, their cultivation, and the benefits that they may provide in improving specific creative thinking outcomes. This broader conceptualization allows for the inclusion of aspects of creative thinking that are more difficult to access in large scale summative assessment environments but may be relevant and attainable for the development of learning solutions as well as summative and formative classroom assessments and tools. The proposed Creative Thinking Learning and Assessment Model defines creative thinking as the capacity to expand beyond conventional boundaries to create unconventional and valuable solutions. Building from the PISA definition given above, this model adds more explicit focus on the importance of first understanding conventionality, its boundaries, and its construction, as a foundation for developing ideas or solutions that can be unconventional and still valuable. Conventionality can be described as the ways in which something is commonly done, or the common solutions for a problem or challenge. We use the term “challenge” to refer to situations that call for creative approaches (e.g., designing a logo) but do not necessarily present problems to be solved. The understanding of conventionality allows for a better understanding of the boundaries and range of unconventionality and supports the processes of generating unconventional solutions. The understanding of conventionality also allows for the evaluation of unconventionality as it relates to the criteria that are required for the unconventional solutions to retain value for the intended purpose. For instance, the lengths of creative artefacts such as songs or movies were originally limited by the materials that were available to record and play them. Identifying that creative artefacts, such as songs and movies, have common lengths, allows for the exploration of why those lengths were common, and whether those factors continue to be limiting criteria. If found to no longer be limiting criteria due to advancements in knowledge, technology, etc., unconventional solutions can be explored that extend beyond those limitations without negatively impacting the value of the creative product. If it is found that those criteria continue to be limitations, creative solutions will need to continue to account for those specific criteria as limiting factors for the evaluation of value.

The model is divided into five creative thinking facets, shown in the circle in Figure 1, that support the skills and processes required for effective creative thinking across contexts, audiences, and domains. We expand on these five facets in this section. Additionally, Figure 1 indicates four essential factors, shown in boxes, that influence creative thinking competence development. The following section provides an overview of those factors and the associated learning and assessment design considerations.

The “Explore” skill is focused on gaining an understanding of the conventions within a domain and/or challenge. The outcomes of exploring might include the identification of conventional features, variables, and boundaries for a domain and/or challenge. This, in turn, includes: inquiring, identifying, understanding, and evaluating the important components of the variables, features, and boundaries around what is considered conventional for both the domain and the challenge (Lucas and Spencer 2017; Baer 2016; Cropley 2006). The intent is that this information is important for understanding the factors for determining what is considered unconventional but also valuable, in that the ideation process is leading toward a novel, viable, and useful product (e.g., an original idea, solution, prototype, artifact, etc.). The key guiding principle in exploration is that problems occurring in real-world environments are rarely well-defined. Instead, most creative thinking occurs when students are working on ill-defined problems characterized by the following: a problem context with boundaries that are not clearly specified; a problem that cannot be solved through routine application of typical experience; and multiple potentially viable paths and different end states. For ill-defined problems, the first stage is identifying and formulating the problem followed by gaining deeper understanding of the conventions within a domain. Another important stage in the creative thinking process is to constantly absorb information from a wide variety of sources in the student’s environment and link new information with existing problems and tasks. This stage requires incubation, which is time to process the information, usually with attention directed elsewhere, allowing for formation of new combinations as a foundation for creative ideas or solutions.

The “Create” skill is focused on imagining and creating new unconventional ideas based on an understanding of what is common or conventional, as described above (Sawyer 2011; Cropley 2006; Runco 2014b). The outcomes of the “Create” skill might include: the creation of multiple solutions that are as different from each other as possible, and/or multiple unconventional solutions that would not fall into categories of solutions previously identified as conventional. The “Create” skill also focuses on the creation of these solutions across a variety of contexts, domains, and/or challenges. This skill also focuses on understanding degrees of unconventionality and how ideation for unconventionality might be influenced and limited by what society and the individual consider to be conventional. Additional focus is placed here on operating within the constraints of what we consider to be valuable, in that this ideation process is leading to a novel solution that meets the value criteria that have been established for the problem or challenge. 

The “Evaluation” skill is used in conjunction with other creative thinking skills throughout the creative thinking process as we seek to both determine what is considered conventional, unconventional, and valuable; and to understand what is influencing those determinations (Sawyer 2011; Cropley 2006). The outcomes of evaluation might include understanding the degrees of conventionality and unconventionality, feasibility, and effectiveness, and the value of a solution, idea, or artifact generated in the creation phase. As Vygotsky (1967) argued: “Any human act that gives rise to something new is referred to as a creative act, regardless of whether what is created is a psychical object or some mental or emotional construct that lives within the person who created it” (p. 7). For that new creation to have value in relation to the problem or challenge, however, the creation must be evaluated against the criteria established for value through the description of the context and reasoning established as the impetus for the problem or challenge. Simple evaluation tasks might ask participants to evaluate ideas against a single criterion; for example, ranking ideas conventionality based on the perceived likelihood that other participants or teams given the same problem or challenge might propose the same idea or solution or the frequency with which an idea was encountered as a solution during the exploration phase. 

More complex evaluation tasks might involve asking a participant to separately rate the conventionality, feasibility (in terms of resources such as time, cost, materials, or effort), and effectiveness (in terms of the qualities or quantities required for the solution to be effective) of each idea in a collection of ideas. As most ideas will not rate highly in all categories, further evaluation will be required to determine which idea, based on the prioritization or balance of those criteria, will allow the participant to identify the idea with the most overall value for the given problem or challenge.

The focus of the “Evaluation” skill is to understand the originality and usefulness of products of ideation and creation in relation to not only the criteria of value established for the problem or context, and in relation to other products, but also in the context of both societal and personal constructions of what is considered conventional, unconventional, and valuable. “Evaluation” might also include an understanding of personal factors, including personal biases and limitations in domain-specific knowledge and experience. “Evaluation” decisions are based on internalized model of the domain and field; these allow for evaluation of novelty and appropriateness. 

The “Improve” skill is focused on further ideation to improve existing ideas and create an optimized solution for the challenge or problem (Lucas and Spencer 2017; Plucker et al. 2004; Beghetto et al. 2014). The outcomes might include the creation of an optimized solution or solutions for the challenge, the improvement of an existing solution based on a new constraint or value criteria, or the modification of a conventional solution to make it more unconventional. The “Improve” skill also allows for the description of the improvements that could be made to optimize a solution for the challenge or problem. The allows for individuals to demonstrate their understanding of improvement without requiring the skill to make the improvement themselves. An example here would be of a food, film, or art critics’ abilities to provide insights for the refinement of a product without requiring the skill to make the actual improvement. This skill utilizes the understanding of what is conventional, unconventional, and valuable, as generated through the other creative thinking skills, as a foundation to revisit and improve existing ideas to create an optimized solution or solutions for the challenge or problem.

Creative thinking in the context of a domain also includes an additional skill: creative communication. The outcomes might include the creation of communication solutions that make information relatable and accessible across a wide range of audiences. For creative thinking to be applied, it is more than just the thought that counts. Successful creators are skilled at predicting how others might react to their novel ideas and solutions and being prepared to respond (Sawyer 2011). Creative communication facilitates the representation and sharing of creative ideas using visuals (e.g., showing instead of telling) and text for the purposes of collaboration or application (Sawyer 2011). The focus is on effectively impacting the reception and understanding of messages across a wide range of audiences by making information relatable and accessible.

## 3. Design Considerations for Learning and Assessment Tasks 

Achieving creative outcomes requires the capacity to engage in creative thinking, but it can also demand a wider and more specialized set of essential conditions, such as domain knowledge, task design, and motivational factors (Amabile and Pratt 2016). 

### 3.1. Domain Knowledge

Whether the creative thinking competency is relatively domain-general (operative over a wide range of settings and disciplines), or relatively domain-specific (operative only in particular domains where the individual has a well-developed knowledge base and a version of the competency adapted to the domain) is a complex and controversial issue. Domains involve an internal, symbolic language; representations; operations on those representations; and a set of everyday practices. A wide range of studies has shown that large portions of creative thinking competency are domain-specific (Sawyer 2011; Baer 1999; Kaufman and Baer 2011; Treffinger et al. 2008). However, there are other perspectives, such as intermediate approaches (e.g., only some traits are domain-general) and procedural approaches (e.g., domain-general skills translate into domain-specific accomplishments). In the proposed framework we adopt the intermediate approach (Plucker et al. 2004; Lubart and Guignard 2004), according to which some creative thinking skills apply to multiple domains (e.g., creating different ideas or taking risks in introducing new ideas), whereas others are unique to specific subject areas (e.g., the ability to create different solutions in science or to write a conceptually different essay in language arts). Although a certain level of domain knowledge is essential for creativity, overly-ingrained, traditional, domain-specific thinking may prevent the individual from manipulating the concepts within a field in novel ways (Beghetto and Kaufman 2014; Barbot et al. 2016). Furthermore, according to Plucker et al. (2004), the level of specificity-generality changes with the social context and as one develops through childhood into adulthood. This approach suggests involving both domain-general and domain-specific dimensions in creative thinking learning and assessment.

### 3.2. Motivation

A person may have creative thinking skills but may not apply them to situations that potentially involve creativity. For example, one may decide to follow other people’s ideas rather than create one’s own, or to decide not to try to persuade other people of the value of these ideas. Even the same task with different instructions (e.g., name as many ideas as possible versus be creative and name as many ideas as possible) can motivate more creative responses (e.g., Forthmann et al. 2016; Nusbaum et al. 2014). Therefore, in order to encourage the decision to be creative, one should believe that he or she will be awarded for the attempt to be more creative rather than punished (O’Hara and Sternberg 2001). In order to promote creativity, there is a need to construct opportunities to engage in it, encourage it, and reward it when people respond to such opportunities. However, most of the conventional assessments penalize students if they try being creative (Beghetto 2010; Smith and Smith 2010). Student answers are often analyzed against prototype responses, while answers that reflect novel perspectives are discouraged. Thus, an educational and social atmosphere in which students feel free to play with ideas is essential for establishing optimal settings for creative thinking learning and assessment. Motivation to be creative can be both intrinsic and extrinsic in nature (Amabile 1989, 1997, 2012). Extrinsic task motivation refers to the external incentives, goals, or pressures that can motivate people to engage in a task. Recent theories, however, have acknowledged that extrinsic motivators such as deadlines or incentives can successfully motivate people to persist in their creative tasks (Amabile and Pratt 2016; Eisenberger and Shanock 2003). Conversely, according to Csikszentmihalyi (1997), people are motivated in creative tasks when they are in an experiential state of *flow*, a peak of intrinsic motivation that is characterized by the key factors such as clear goals; a high degree of concentration on the task; a loss of self-consciousness; a distorted sense of time; immediate feedback; balance between level of ability and the challenge of the task; a sense of personal control; and the focus of awareness being narrowed to the activity itself, so the action and awareness are merged. When in flow, students are in the optimal position to demonstrate their creative thinking.

### 3.3. Facilitation

To be creative, one must first decide to generate new ideas, analyze these ideas, and share the ideas with others. Cultivating a supportive climate for creative thinking is key to encourage learners to engage effectively in creative thinking and sustain the effort over time (Beghetto and Kaufman 2014). Facilitation and feedback provided by teachers are reinforced by teachers’ shared belief that creative thinking skills can be cultivated in the learning environment, and the development of the skills reinforces students’ domain knowledge. Teachers’ understanding of the importance of students’ idea diversity, risk-taking, and multimodal means of creative expression play a major role in creating a supportive climate. Nickerson (2010) proposed a set of rules that can stifle creative thinking in classrooms as a form of warning to teachers hoping to foster creativity: (1) tolerating no deviation from the one correct method expected from the learners and using only one correct answer to a question; (2) promoting unquestioning and fear of authority and tolerating no changes in perspectives or solutions; (3) adhering to prescribed steps or sequence of activities at all costs; (4) promoting a narrow view on creative thinking aligned with Big-C and cultivating the belief that originality is an extremely rare quality; (5) discouraging students from making interdisciplinary connections and applying knowledge or problem-solving approached from one domain in another; (6) discouraging curiosity and inquisitiveness; (7) promoting the belief that creativity is an unchanging property of an individual; (8) and above all, never permitting learning, problem solving, and creative expression to be fun. Creativity researcher*s* have also emphasized the importance of monitoring the motivational messages to students. Focusing students’ attention on interesting and personally meaningful aspects of tasks and encouraging self-improvement, increased effort, a growth-oriented mindset, and seeking help from others when necessary are among the key recommended facilitation practices (Beghetto 2010; Beghetto et al. 2014; Karwowski 2014). 

Teachers advance creative thinking when they ensure a climate of psychological safety. Psychological safety is achieved by accepting and valuing all students’ contributions; stressing cooperation among students; limiting time constraints and competition; and fostering students’ autonomy (Amabile 1989; Treffinger 2005). Teachers who facilitate creative thinking activities fulfill a range of roles, such as that of the coach, domain expert, and cheerleader (Newman 2006). The ultimate support system includes detailed teaching guides, ongoing professional development, and active teacher engagement in the development of learning and assessment activities, which is an integral part of a creative thinking program. 

### 3.4. Task Characteristics

Task-specific considerations for creative thinking learning and assessment require tasks to be authentic and represent a wide range of domains (e.g., science, social sciences, math, language arts) and situations (e.g., personal, public, educational, occupational). Creative thinking tasks might overlap across domains and situations as well as means of expression (e.g., visual, written). Cultural diversity, fairness, and sensitivity considerations (e.g., religion, politics, violence, racism) are among further considerations in the design of creative thinking tasks. 

Designing tasks to make the otherwise implicit processes involved with creative thinking more explicit requires the creation of new item types and scenarios designed to support skill development. We have developed sample task models to explore the potential for formative assessment of creative thinking skills and competencies. Sample task models illustrate the conceptual ideas of the framework, ensure that those ideas are functional with the target population, and validate the core ideas for the task models and item-types. These task models were developed based on the integration of a well-established approach to assessment, evidence centered design (ECD) (Kim et al. 2016), with a universal design (UD) approach to learning (Rose and Gravel 2009). Combining these approaches increases the likelihood that learning and assessment activities will be well aligned with the competency model and targeted knowledge areas, while being accessible to all students, including students with disabilities. The advantage of following an integrated ECD and UD principled design is particularly evident when the goal is to develop and assess complex competencies, such as creative thinking by using complex multi-step performance tasks. It is important to explicitly identify how the relevant competencies and behaviors are connected because the complexity of the focal competencies, and/or the rich data the tasks provide might pose difficulties in making inferences from behaviors to competencies. 

ECD formulates the process of assessment development to ensure consideration and collection of validity evidence from the onset of the assessment design (Kim et al. 2016; Mislevy et al. 2006). ECD is built on the premise that an assessment is a measurement instrument, with specific claims about the test scores are associated, and that a good test is a good match of test items and test takers’ skills. The ECD principles define several interconnected models, including a competency or student model, an evidence model, a task model, and an assembly model. Using ECD as an organizing framework for learning and assessment can help to address a series of important design questions; namely: Which constructs or processes does each task within the learning or assessment reveal? Do the proposed scoring methods effectively recognize and interpret the evidence generated by students’ responses and interactions? How is all the evidence that is generated by students’ choices synthesized across multiple tasks? Is all the evidence for a construct comparable when different students attempt different tasks? In a recent study, Arieli-Attali et al. (2019) described an extension to the ECD framework (termed e-ECD) such that it includes the specifications of the relevant aspects of learning at each of the three core models in the ECD, and it makes room for specifying the relationship between learning and assessment within the system. The proposed framework does not assume a specific learning theory or learning goals, but rather allows for their inclusion within an assessment framework, such that they can be articulated by researchers or assessment developers that wish to focus on learning. 

Measurement models, combined with the learning sciences’ perspective in the development of learning and assessment activities, provides the essential foundation for curriculum-embedded creative thinking models in the future. The latent competencies that are articulated and defined in the creative thinking competency model establish the conceptual basis of the learning and assessment system, and they are often based on a theory or previous empirical research related to learning objectives (e.g., foster the ability of students to generate original solutions in the context of scientific problem solving) and assessment goals (e.g., valid and reliable measure of the ability of students to evaluate and improve solutions in the context of scientific problem solving). Since we cannot tap directly into the latent competencies, we need to design activities/tasks such that they will elicit behaviors that can reflect on or indicate about the latent competencies. The task model specifies the task features that are supposed to elicit the observables, and only them, such that to allow inferences about the latent competencies. For example, if the assessment is intended to measure the “Create” skill, focusing on the ability of the student to develop a wide verity of unconventional solutions, the tasks should be designed with care such that reading ability and the tools available for the students are not an obstacle to perform well on the task and express one’s skill to generate unconventional solutions. ECD principles can be flexibly adapted across different learning and assessment contexts of creative thinking. Although we focus on ECD here, alternative frameworks that follow a principled approach for learning and assessment design can also be considered (e.g., Luecht 2013; Nichols et al. 2016).

UD emphasizes the importance of addressing accessibility for the broadest range of potential users during the initial stages of learning and assessment design and throughout the development and implementation of the assessments (Rose and Gravel 2009). The use of UD principles creates flexible solutions because from the start designers consider the diverse ways in which individuals will interact with the learning and assessment activities. When sources of construct-irrelevant variance are identified using an ECD approach, the application of UD principles can guide the incorporation of appropriate options for how students interact within the system. In this way, ECD works synergistically with UD. By considering multiple means of perception, expression, cognition, language and symbol use, executive functioning, and engagement, the application of UD in the ECD process accounts for individual differences in how students recognize, strategize, and engage in learning and assessment situations. Providing multiple means of creative expression and the ability to use a wide variety of tools and modalities in support of creative problem solving are among the key essential conditions for creative thinking. This synergistic process of combining ECD and UD principles minimizes the potential unintended negative influence that accessibility needs may have on student performance and maximizes the opportunities for students to show what they know and can do.

## 4. Task Models 

In this next section, we describe the key considerations for the design of creative thinking tasks and task models. The development of a formative assessment relies on specific, validated models of assessment that can be applied at scale. One of the central goals of this paper is to build from prior models and frameworks to integrate the key ideas into a unified approach to formative assessment. To this end, task models (a component of the e-ECD framework) are needed to specify how evidence of creative thinking can be elicited from students. We choose two sample task models to illustrate the integration of the skills and considerations for assessment described in the preceding sections. 

Conventional domain-general tasks have been used effectively to target isolated demonstrations of creative thinking skills, such as convergent and divergent thinking, in a way that allows for their effective measurement (Runco 2014b; Torrance 1966). Building on this valuable understanding, novel task models are being designed to allow for both the learning and measuring of these skills with improved authenticity, engagement, and effectiveness. These task models focus on tools, functionalities, and environments that allow us to replicate the real-world scenarios and processes involved with creative thinking. These new environments allow participants to explore the skills in more authentic environments in which the creative thinking skills are being used in context, in concert, and in a coordinated way, to create novel, ideas solutions, and artefacts. These new environments are also intended to facilitate the learning and formative assessment of creative thinking skills. By moving from formal and familiar assessment environments in which students are traditionally encouraged to seek expected and correct solutions, following a set of linear series of processes, the intention is that these new environments will allow for and encourage students to move beyond a mindset in which the expected and correct solutions are preferred, to an environment in which they are encouraged to think more broadly, flexibly, and outside of the conventions of traditional expectations. The intention is that these novel environments will reflect the dynamic, non-linear, adaptable, and iterative ways in which the creative thinking process occurs in real-world creative thinking experiences and across a range of domains (Mumford and McIntosh 2017; Botella and Lubart 2019). The intention is also that these dynamic and novel environments will enhance the motivation of participants, improving the learning and measuring of creative thinking skills (Rosen and Tager 2013) and the likelihood that the skills will transfer to real-world applications (Beghetto et al. 2014; Kaufman and Baer 2011). Perhaps most importantly, these novel task environments allow for expanded opportunities for participants to learn and demonstrate their creative thinking skills. The engaging functionalities and tools involved with these task models open the doors for successful learning and demonstration of these skills to participants that are more adept at “showing” than “telling,” in the visual sense. They also open the door for participants who are more adept at “telling” through a contextualized dialog or conversation wherein the creative thinking process is providing the environmental context than “telling” through the selection of a response in a decontextualized context wherein the skill is being explored in isolation and outside of the process in which it will be applied.

Two task models that capitalize on the advantages of these novel assessment and learning environments are the conversational task model and the visual representation task model. Each of these task models allows for exploration of the creative thinking process in context and with tools that expand beyond conventional interactive, simulation-based, and basic constructed and selected-response task models. Previous studies indicated promising results in the context of psychometric properties of these tasks models (Rosen and Tager 2013; Rosen 2017; Rosen et al. 2018, 2019). However, further research is needed to establish more comprehensive evidence on these task models across domains and contexts. 

### 4.1. Conversational Task Models

In real-world contexts, the creative thinking skills that lead to a novel solution are often experienced through a series of steps where the processes and skills involved are implicit rather than explicit. For example, in real-world contexts, novel solutions to problems or challenges may be reached through brainstorming conversations with the right person or group of people. Group collaboration is often noted to be more effective than individuals at creating novel solutions to complex problems (Baer 2016; Warhuus et al. 2017). The essential steps for creative thinking can be accounted for within this conversational process, although they might not be explicit or apparent at the time. The use of conversational task models allows for the replication of group creative thinking process in a way that makes those implicit processes and skills explicit. 

This model facilitates our ability to explore, support, remediate, teach, and measure these processes and skills both in real-time and in an authentic real-world context. Conversational task models also allow us to cultivate and measure these skills with an enhanced ability to address factors impacting authenticity and propensity, since the task itself is an accurate representation of the creative thinking process (Botella and Lubart 2019; Rose et al. 2000). The intention is that when the skills are being elicited in an environment in which they are most likely to be demonstrated in a real-world scenario, and in which the participant is being engaged in a personally meaningful way, demonstration of the skill is more likely to reflect the participants actual proficiency with that skill as it relates to creative thinking (Beghetto et al. 2014). Similarly, these environments offer us not just a better understanding of the proficiency with which the skill is demonstrated, but also the propensity to demonstrate the skill, or the likelihood that the student will engage in the task in an authentic and invested way (Amabile 2012; Zhou and Su 2010).

Conversational task models utilize tools that allow us to replicate conversations (dialog) between two or more actors/agents where the learner contributes as an active participant. In conversational task models, the learner engages in dialog with virtual agents that can represent a range of peers, experts, and advisors (Graesser et al. 2017; Rosen 2017). In a conversational task, the virtual agents and the participant engage in a series of conversational exchanges that are most often represented as a series of ‘chat’ exchanges. The contributions of the virtual agents to the conversation are pre-scripted and are designed to replicate a conversation flow that will routinely prompt the participant for a response. The participant’s responses to the virtual agent’s prompts can take a variety of forms depending on the skill or process being learned and/or measured. Responses range from the option to choose from a pre-scripted set of responses that have been designed to align with the varying proficiency levels of a specific skill, such as identifying, ranking or rating options representing a range of conventionality and features related to conventionality; to constructed responses, which provide an open field for the participant to contribute their response, such as those involved in brainstorming or the evaluation of conventional and unconventional ideas. Pre-scripted responses are aligned with levels of skill proficiency to facilitate the ability to efficiently, effectively, and with an optimized degree of reliability and validity, measure levels of skill. Constructed responses can be used to explore processes such as brainstorming, or as an open field for contributing unconventional ideas or solutions. Constructed responses are also utilized for evaluating or iterating on ideas, contributions, or even multi-media artefacts presented by virtual agents within the task. 

One advantage of this type of dialog is the ability of conversational task models to explicitly draw out the intrapersonal factors influencing the evaluative skills required for creative thinking (cf. Ge and Land 2004). This further enhances the value of this task type in that students can be asked to expand on, explain, or provide reasoning for their responses within the chat environment, based on their selected or provided response. The evaluative aspects of the conversational task model prove particularly useful in the exploration of reasoning and biases that might influence a participant’s understanding of conventionality in the same way that these aspects might realistically be explored through conversation during the real-world creative thinking process that occurs within a group setting. 

Conversational task models also introduce the opportunity to include real-time feedback for participant responses. Feedback is inherent in an authentic conversation, and a valuable component of effective creative thinking in a group setting, particularly for processes that involve brainstorming, iterating, and the evaluation of ideas. Feedback from advisors, mentors, and peers in the conversational task model can fulfill a range of purposes valuable for creating an environment that encourages creative thinking, including the ability to provide encouragement, remediation, scaffolding, and the solicitation of insights into participants’ reasoning and reflection during creative thinking processes (Cropley 2006; Tighe et al. 2003; Zhou and Su 2010). 

Feedback in the conversational task model can take a variety of forms. Pre-scripted feedback can be designed to align with specific pre-scripted response options or built into the chat in a way that supports the conversation flow. For example, pre-scripted feedback can be used to represent encouragement from virtual peers, experts, and advisors for high-level skill selections or to provide remediation and support for low-level skill choices, improving engagement, motivation and supporting deep learning (D’Mello and Graesser 2012; Ge and Land 2004). Feedback in pre-scripted environments can also be used to elicit additional information from students regarding their thought processes, supporting understanding of metacognitive processes and the reasoning behind selected responses. By providing engaging extrinsic feedback, these virtual agents can support the convergent processes of creative thinking such as evaluating and selecting ideas, as well as the divergent processes of creative thinking such as brainstorming and iterating on ideas (Collins et al. 1999). The inclusion of peer feedback, as opposed to teacher or advisor feedback, may also reduce the stress of evaluation, which is known to contribute to an environment in which students might feel limited in their motivation to engage in the creative thinking process (Stipek 1998).

The pre-scripted conversational task model environment provides a range of additional benefits related to authenticity and propensity as well. The pre-scripted environment allows for optimized control over the context in which the participant is experiencing the creative thinking task and demonstrating the processes and skills. This allows for the control of factors that could influence the ability of students to explicitly address specific creative thinking processes and skills. For example, the pre-scripted environment can allow for the strategic inclusion of required knowledge, information, and resources required for completion of the task, mediating the influence of prior knowledge. In a task where participants are asked to brainstorm a short list of the common features of a specific entity, curated collections of multimedia elements chosen to highlight those common features can be included to support, and if necessary, scaffold, the identification of the common features. This facilitates a common understanding among participants as the task moves forward and common set of conventions for scoring. In a task where background knowledge of the problem or challenge would typically be provided as a stimulus to be reviewed from the outset and then remembered until required for application of the task, the conversational task model allows for this information to be revealed and included in the part of the process in ‘real-time’, when the information is most relevant in the context of the task, and when that information would most likely be shared in a real-world context. The knowledge elements included during these pre-scripted encounters can then be used as a foundation for task-specific activities with the understanding that all participants are able to interact with the task with a common understanding. Additionally, virtual agents such as peers, experts, and advisors can also be designed to represent a wide range of perspectives as they can be historical/fictional, novice/expert, peer/advisor, supportive/challenging, demographically neutral or designed to intentionally elicit bias. The pre-scripted conversational task model environment also allows for the inclusion of design elements that can expand the context beyond those available in human-to-human conversation to include the immersion of the participant into historical and fictional contexts. 

**Figure 2:**
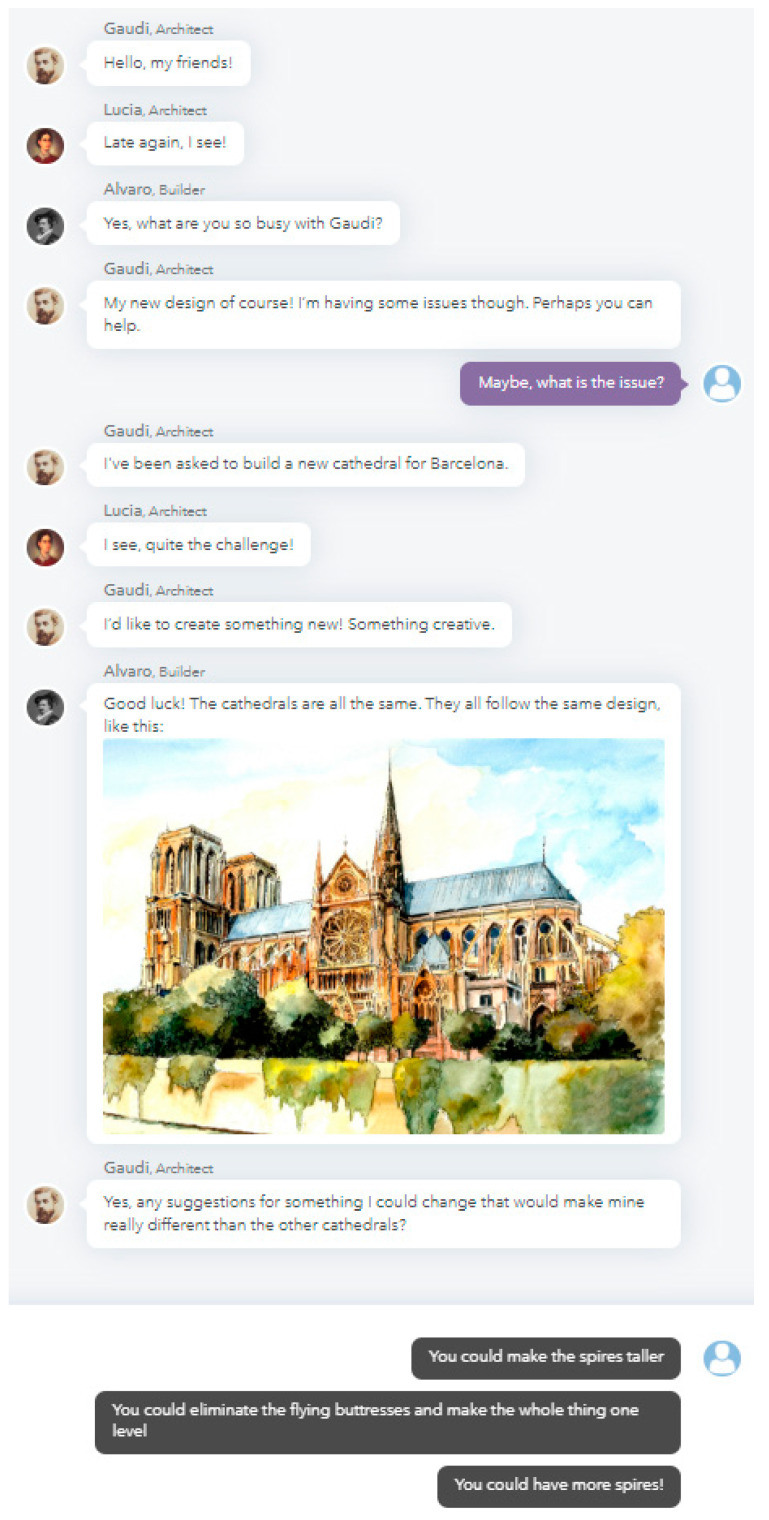
Illustration of the conversational task called Architecture Design Challenge.

One example of our conversational task model is a task called Architecture Design Challenge, shown in Figure 2. The Architecture Design Challenge is a conversational task model used to replicate the creative thinking process occurring as an historical figure, Gaudi (i.e., virtual Antoni Gaudi), explores ideas for novel, innovative design. Participants are asked to join in the conversation with a builder and architect to contribute to the process of identifying what features are common to current designs of the time and what features the new design could include that would set it apart. The conversational task model encourages participants to interact with virtual Gaudi, the architect, and the builder to brainstorm common features of schools that had been or were being constructed at that point in history and explore unconventional ideas for features that could be changed or added. Gaudi, and the others in the chat, can provide visuals and multimedia examples of designs from the time and encourage the participant to help them identify some of the common features of those buildings. The chat proceeds to explore the reasoning for why those features might have been commonly used and whether those reasons continue to apply as constraints or whether those features are good candidates for exploration in terms of new approaches to their new, creative design or application. With encouragement and feedback from the group the participant is led through a process that allows them to identify new, unconventional ideas, iterate on those ideas, and evaluate those ideas for value and viability in terms of their inclusion in the new design. Multiple choice response options in the chat are designed to reflect full, partial, and no-credit responses for the skill being assessed (Rosen 2017). Responses chosen by the participant in the chat can then be auto-scored. Open-ended responses can also be included and human-scored, allowing open creative idea generation and brainstorming processes to be included. These items allow the conversational model to support assessment, practice, and learning processes. Open-ended responses can also be included to collect ideas that can be used to curate and update future version of the task, refine response options, and improve the scoring model. Response scoring ranges from 1 to 10 indicate no, partial, or full credit at each dialog node. As students progress through the conversational task and complete nodes related to each skill, skill-level scores accumulate and range from 0%–100% (as reported to students). Skills can be weighted differentially, as noted above, in which case higher-weighted skills contribute more to an overall task score. The overall task score also ranges from 0%–100%, as reported to students and teachers in addition to the skill-level reporting. Real-time reporting is supported by embedded automated scoring as each response is selected. The chat environment also allows for the inclusion of materials to be provided with the scoring to provide actionable definitions of the skills and scoring designed to facilitate insights beyond a “number” score (Rosen et al. forthcoming). 

### 4.2. Visual Representation Task Models

The creative thinking skills that lead to a solution that expands beyond conventional boundaries sometimes require methods of communication and collaboration that expand beyond traditional boundaries as well (Sawyer 2011). Due to the abstract nature and form that creative ideas, responses, processes, and solutions may take, it is sometimes easier and more efficient to “show” than it is to tell. Despite this, visual tasks have been relatively less common than verbal tasks in the literature (Kim 2005).

Tesla wrote in a 1919 essay (Cheney 2011): “When I get an idea I start at once building it up in my imagination. I change the construction, make improvements and operate the device in my mind” (p. 12). It may be easier to sketch out a new design than to create the image of the design using imagination or describe that design using text. It might also be easier to illustrate a complex process using a flow chart than to provide a description of the processes, their dependencies, and the critical relationships contained within those processes. The potential of creative thinking when applied in real-world contexts to bring together fields and experts will also require the ability to transcend the challenges related to communicating and collaborating effectively not only between fields and domains of knowledge, but also across languages and cultures. As such, the ability to use visual representations to support collaboration and communication in the creative thinking process is becoming increasingly important. This is just one example of the potential value for the use of task model environments that allow participants to express their ideas visually and to explore creative thinking skills in more authentic environments in which they are being used in context, in concert, and in a coordinated way, to create unconventional ideas, solutions, and artefacts.

Visual representation task models allow students to demonstrate creative thinking skills through the creation of visual artefacts. These artefacts are created using visual design tools that allow students to use a range of elements to communicate their ideas without exclusively using text. These design tools incorporate a curated collection of tools, images, and functionalities that allow participants with a wide range of proficiency with visual expression tools to demonstrate their ideas. For example, stamp libraries and basic design functions to create and adjust the size and color of shapes, lines, and text can provide a common set of tools to help mediate the gap between students with low versus high proficiency/experience with design tools. Graphical elements to create flow charts that express processes and connections or to represent data (pie charts, graphs, etc.) can also be incorporated into the design tools to facilitate and scaffold the visual artefact creation process. Although the intent is for students to express their ideas without the limitations of exclusively using text, we also include a “text tool” in the design environment to allow inclusion of text descriptions to enhance their ability to relay the “visual message” to the intended audience. This also reflects the real-world use of visual and graphical elements to communicate ideas effectively.

One of the most important functions of the design tool environment is to facilitate learners’ demonstration of their understanding of the features that define and create the boundaries for conventionality as they relate to the creative thinking process. By utilizing the design environment for the creation of both conventional and unconventional artefacts participants are enabled to “show” rather than “tell.” Learners can make visual connections and illustrate relationships between features and ideas. The spatial aspects of the design environment also allow for the illustration of evaluative concepts such as value, and degrees of qualitative differences in an illustrative way that facilitates understanding. For example, a visual tool will allow a learner to relay not only the ranking or order of ideas in terms of conventionality but also the degree to which those relate to each other on the spectrum of conventionality.

An additional advantage of visual representation task models is the potential to include tools that allow for the inclusion of multi-media artefacts, where students will be able to create, upload, or otherwise include multimedia elements such as video and audio artefacts. These expanded opportunities come with additional complexities for scaling, involving considerations such as technology requirements and the range of familiarity and proficiency among students with the tools used to create multi-media, as well as added complexity for scoring procedures. Other visual tools, such as galleries of objects and images depicting visual representations, can be included as well. For example, tasks that incorporate the use of galleries of objects and images might ask students to evaluate a collection of images created by other participants to represent a conventional object or image and identify the common features and further rank those features on a scale of common to uncommon (cf. Wilken et al. 2019). These steps use visual representation within the creative thinking process to encourage participants to understand not only the elements of conventionality, but also to understand their value in relation to each other as well as the intended solution for the problem or challenge. 

One example of a challenge that draws upon a range of visual representation task models is a task called AI-Spy. The AI-Spy task utilizes visual representation tasks including design tools and visual gallery environments to explore the design and evaluations of visual artefacts that are intended to either be either recognized by an AI designed to recognize specific visual artefacts or to evade an AI designed to recognize these visual artefacts. In the task, participants use a design tool to create a specific object or idea, or other familiar object or figure, so that an AI tool designed to recognize images of that specific object or figure will be able to identify the participant’s creation. Participants then identify which visual elements they included to ensure that the AI would recognize the figure (e.g., whiskers, pointy ears, cat eyes, long tail). These steps use visual representation tools to allow participants to engage in the creative thinking thought processes designed to help participants identify what are broadly considered conventional or common features. Next, participants design an image of a cat that will not be identifiable by an AI tool designed to recognize images of cats, but that a human will still be able to identify. Participants include their reasoning for the elements and features that they chose to include. These steps use visual representation tools to encourage participants to create an unconventional artefact that exhibits the “value” necessary to allow an audience to recognize an unconventional representation of a familiar object or figure. Participants are asked again to describe their reasoning for the elements that they chose to include. Finally, participants are presented with a conventional representation of a cat and then iterate on or modify the design to create a final design that would again be recognized by humans, but not by an AI tool designed to recognize images.

## 5. Discussion

The new focus on nurturing creative thinking in educational systems poses many new opportunities both for researchers and practitioners. Defining creative thinking competency within the context of changing curricula worldwide and developing effective teaching, learning, and assessment practices in schools are among the key opportunities. The definition of creative thinking competence goes beyond the traditional focus on divergent thinking; it also requires evaluative and convergent thinking, as well as a great deal of domain knowledge and skills. Fostering creativity in schools and teaching specific content knowledge need not be in opposition, as often feared by educators. Creative thinking requires significant knowledge of conventions within domains, and applying creative thinking to real-life challenges helps deepen one’s knowledge on the related topics. Many creative thinking skills, such as exploration and improving, can be used to foster both creative thinking and knowledge of specific content. This paper summarizes a research-based conceptual framework for research and development of ways to foster and measure creative thinking in schools. The paper provides an overview of the literature to define observational behaviors and an operational definition of creative thinking competency in students, and illustrate how the environments and tasks for the nurturing creative thinking may lead to further empirical work and may contribute to the design and development of creative thinking learning and assessment in schools. We describe our own design and development work with respect to the possibility of encouraging the growth of creative skills and assessing them within digital environments, in conjunction with academic subjects. 

Task models, such as simulations and those requiring simple written and selected responses continue to be valuable for cultivating creative thinking skills (Lucas and Spencer 2017; Rosen and Tager 2013). Tasks requiring text-based responses allow students to quickly and effectively engage in creative thinking processes, such as brainstorming, and to engage more deeply in creative thinking processes, such as those involved with the creation of longer texts required to generate and communicate creative ideas. Student familiarity and comfort with written tasks may support an environment that allows them to engage in the creative thinking processes, particularly those involving divergent thinking, in a manner that positively impacts their ability to generate unconventional ideas (Runco 2014b). Expanding on these familiar task models, new task models and environments allow for and encourage students to move beyond a mindset in which the expected and correct solutions are preferred to an environment in which they are allowed to think more broadly and outside of the conventions of traditional expectations, which can limit creativity (Amabile 2012; Rosen et al. 2018; Runco 2014b; Tighe et al. 2003). The utilization of these new task models also allows participants to explore skills in more authentic and immersive environments. This allows students to learn and demonstrate creative thinking skills in environments that use these skills and processes in context, in concert, and in a coordinated way, to create novel ideas, solutions, and artefacts, potentially improving the transfer of creative thinking skills to real-world tasks. 

Future research in this area needs to be methodologically sound and transparent, so should provide clear evidence of the effectiveness of learning environments aimed to foster creative thinking. It is important to undertake research which explores the links between the effectiveness of creative thinking approaches and diversity of the students across different countries, cultures, languages, and background variables, such as socio-economic status. Research designs that capture the baseline prior to the introduction of creative thinking learning might help in establishing this evidence clearly. Further research is required that can clearly establish the interrelationship of creative thinking learning environments and students’ knowledge in academic domains, motivation, and social-emotional skills. Longitudinal studies should be undertaken that can identify the long-term impacts of creative thinking environments in schools across school years and ages. In summary, although illustrative in purpose, the work described in this paper was intended to contribute to the growing literature on learning and assessing creative thinking in school settings.
